# Effectiveness of the Walking in ScHools (WISH) Study, a peer-led walking intervention for adolescent girls: results of a cluster randomised controlled trial

**DOI:** 10.1186/s12966-024-01563-0

**Published:** 2024-02-19

**Authors:** Marie H. Murphy, S. Maria O’Kane, Angela Carlin, Ian M. Lahart, Leanne C. Doherty, Russell Jago, Gary McDermott, Maria Faulkner, Alison M. Gallagher

**Affiliations:** 1 Centre for Exercise Medicine, Physical Activity and Health, Sports and Exercise Sciences Research Institute, Ulster University, Belfast, BT15 1ED UK; 2 Physical Activity for Health Research Centre (PHARC), Institute for Sport, Physical Education and Health Sciences, University of Edinburgh, Edinburgh, EH8 9YL UK; 3 Institute of Nursing and Health Research, Ulster University, Derry/Londonderry, BT48 7JL UK; 4 Faculty of Education, Health and Wellbeing, University of Wolverhampton, Walsall, WS1 3BD UK; 5 Population Health Sciences, Bristol Medical School, University of Bristol, Bristol, BS8 2PL UK; 6 Sports Lab North West, Atlantic Technological University, Letterkenny, Ireland; 7 Nutrition Innovation Centre for Food and Health (NICHE), Biomedical Sciences Research Institute, Ulster University, Coleraine, BT52 1SA UK

**Keywords:** Physical activity, Walking, Adolescent girls, Peer-led, School-based, Intervention, Low-cost, Behaviour change

## Abstract

**Background:**

Most adolescent girls fail to meet current physical activity guidelines. Physical activity behaviours track from childhood into adulthood and providing adolescent girls with opportunities to be physically active may have health benefits beyond childhood. The effects of walking interventions on adult cardiometabolic health are known, however less is understood about the potential of walking to promote physical activity in adolescents. Following the Walking In ScHools (WISH) feasibility study, this definitive trial aimed to evaluate the effectiveness of a novel, low-cost, school-based walking intervention at increasing physical activity levels of adolescent girls (aged 12–14 years).

**Methods:**

Female pupils were recruited from eighteen schools across the border region of Ireland and in Northern Ireland. In intervention schools (*n* = 9), girls aged 15–18 years, were trained as walk leaders, and led the younger pupils in 10–15 min walks before school, at break and lunch recess. All walks took place in school grounds and pupils were encouraged to participate in as many walks as possible each week. The primary outcome measure was accelerometer determined total physical activity (counts per minutes, cpm).

**Results:**

In total, 589 pupils were recruited to the study. At baseline, pupils engaged in a median (interquartile range (IQR)) 35.7 (21.2) mins moderate-vigorous physical activity (MVPA) per day and only 12% (*n* = 66) of participants met physical activity guidelines (60 min MVPA per day). The intervention was delivered for a mean (standard deviation (SD)) 19.9 ± 0.97 weeks. The mean post-intervention total physical activity for the intervention group was 676 cpm and 710 cpm in the control group. Post-intervention total physical activity did not statistically differ between groups when adjusted for age, body mass index z-scores and baseline physical activity (mean difference, -33.5, 95% CI = -21.2 to 88.1; *p* = 0.213).

**Conclusions:**

‘Scaling-up’ physical activity interventions is challenging and despite a promising feasibility study, the results of this fully powered trial suggest that in this context, the WISH intervention did not increase device measured physical activity. Since the COVID-19 pandemic, school environments have changed and although pupils enjoyed the programme, attendance at walks was low, indicating that there is a need to better understand how to implement interventions within schools.

**Trial registration:**

ISRCTN; ISRCTN12847782; Registered 2nd July 2019.

**Supplementary Information:**

The online version contains supplementary material available at 10.1186/s12966-024-01563-0.

## Background

Regular physical activity in adolescence is associated with beneficial health outcomes, including a reduced risk of obesity, improved physical fitness and cardiometabolic health, increased muscle and bone strength and reduced risk of depression [1–4]. Despite this, it is estimated that globally 81% of adolescents (aged 11–17 years) fail to meet the World Health Organisation (WHO) recommendation of 60 min of moderate to vigorous physical activity (MVPA) per day [5, 6]. Recent data from the International Children Accelerometry Database shows that girls typically participate in less physical activity (43 min MVPA per day) than boys (63 min MVPA per day) [7] and less girls (15%) than boys (22%) meet physical activity guidelines [5]. In addition it is recognised that the gap in terms of the prevalence of insufficient physical activity (< 60 min MVPA per day) between boys and girls has widened over time in some high income countries including Ireland [5]. The latest data from the Children’s Sport Participation and Physical Activity (CSPPA) Study which was conducted across the Island of Ireland reports that twice as many post-primary school girls (22%) were inactive (60 min of physical activity on 0–2 days per week) when compared to post-primary boys (11%) [8]. Physical activity levels decline as children move into adolescence and through to adulthood [9–11]. In a UK longitudinal study [12], it was reported that time spent in MVPA decreased by 12 min per day from age 10 to 14 years. Furthermore, research estimates that there is a 4.2% average annual reduction in total physical activity from ages 5 to 18 years and that this decline is most pronounced among adolescent girls (4.6% in girls versus 3.7% in boys) [13]. Addressing low levels of physical activity among adolescent girls is a public health priority particularly as physical activity habits adopted during adolescence may track into adulthood [14, 15] and increase the likelihood of developing many chronic health conditions including cardiovascular disease, obesity and diabetes mellitus [16].

The Global Action Plan on Physical Activity recommends that opportunities for physical activity should be integrated across multiple settings including schools [5]. Schools are an excellent setting for physical activity promotion [17] and can reduce health inequalities [18]. Virtually all children in the UK and Ireland attend school and it is estimated that for children, 40% of their waking time is spent at school [19]. Often schools have space, resources and staff to deliver health promotion initiatives [20]. There are several opportunities for children to be physically active across the school day including, during breaktimes, active travel to and from school, classroom-based physical activity, and Physical Education (PE) classes [21]. However, there is a lack of consensus on how to best promote physical activity within the school setting [17]. School-based Physical Education (PE) provides young people with the opportunity to participate in structured physical activity and develop their skills, competence, confidence and motivation [22, 23]. Post-primary schools across the island of Ireland are recommended to provide 120 min of PE per week [24, 25], however, only 23% (Ireland) and 40% (Northern Ireland (NI)) of pupils receive this amount of PE [26]. Most schools rely on PE programmes to provide adolescents with the skills and motivation to participate in physical activity, however, adolescent girls typically have lower PE participation rates [27] and fewer opportunities to be physically active during the school day than boys [26, 28, 29]. Previous school‐based interventions have had limited effects on physical activity, and have not led to sustainable behaviour change [30–33]. Concurrent with mounting workloads and increased pressures (for example, finance and student achievement) on teachers [34]*,* there is a growing interest in the potential of peer-mentoring based interventions to influence health related behaviours [35–37].

Peer-led interventions have been shown to increase physical activity in several population groups [38] including adolescents [39]. The underpinning mechanism can be explained by Social Cognitive Theory whereby individuals learn behaviours by observing and imitating others [40]. Peer-mentors are an important component of many intervention delivery models [30] and can provide various types of support including motivation and encouragement [38, 41]. They are seen as a role model for younger people and are perceived to have a better understanding of their needs [30]. Peer-mentors can empower younger people to participate in regular physical activity by strengthening their social network and increasing self-efficacy [30, 42].

Self-efficacy, social support and motivation are important considerations for physical activity promotion among females [43] and self-determination theory has emerged as a popular framework for examining the relationship between motivation and physical activity [44]. Another important consideration for the development of effective interventions for adolescent girls is perceived competence [45]. During adolescence, a lack of interest in the physical activities offered in school [46], and a focus on team-based, structured sports [47] are less likely to engage girls [48]. Walking has been described as the most natural form of physical activity, it requires no specialist skills to participate [49] and addresses many of the reported barriers to physical activity including a perceived lack of time, equipment, skills and competence [50]. A meta-analysis by Murtagh et al. (2015) reported that walking increased aerobic capacity and reduced blood pressure, waist circumference, weight, percentage body fat and body mass index (BMI) among adults [51], however less is understood about the effects of walking in adolescents. Walking interventions, particularly those implemented in a school-setting may have the potential to increase physical activity among adolescents [52], and in particular among girls who are less active and prefer non-competitive and less-structured physical activity [53, 54]. The potential of walking interventions to increase the proportion of adolescents meeting physical activity guidelines is largely unknown. One study by Lee et al. (2012) reported that an intervention whereby adolescents were provided with pedometers and instructed to complete 12,000 steps and/or 60 min aerobic walking/day and set individual goals had no effect on the percentage of pupils meeting physical activity guidelines [55]. However, in other studies where walking interventions were implemented, the percentage change in the percentage of pupils meeting PA guidelines was unreported [56–58].

Physical activity interventions delivered at school but outside of curriculum time are likely to be more acceptable as schools are under increasing pressure to improve academic standards and performance [59]. In recent years, there has been growing interest in the promotion of physical activity during school recess [60], which may be an opportune time to encourage physical activity for adolescent girls who are less active during recess compared to boys [60, 61]. However, despite the potential of school recess to promote physical activity, there is a lack of intervention research in adolescent girls [62] and given the importance of increasing physical activity among adolescent girls, finding effective, sustainable, low-cost interventions is essential.

The aim of this cluster randomised controlled trial (c-RCT) was to evaluate the effectiveness of a novel, low-cost, peer-led school-based walking intervention, delivered across the school year, at increasing physical activity levels of adolescent girls in Ireland and Northern Ireland (NI). It was hypothesised that if pupils took part in multiple short walks across the school day, time spent in MVPA would increase, an increased proportion of pupils would meet current physical activity guidelines and if sustained, there would be a meaningful effect on health outcomes.

## Methods

### Study design

The study design was a two-arm c-RCT to compare the Walking In ScHools (WISH) low-cost, peer-led, school-based walking intervention against usual practice control with an embedded process evaluation. The study protocol was published in 2020 [63] and the trial was registered prospectively with ISRCTN (ISRCTN12847782) in July 2019. The study design was informed by a feasibility pilot study [58] that used the Medical Research Council (MRC) [64] framework for complex interventions to develop a school-based, peer-led walking intervention. The feasibility study was designed following an iterative process and was informed by a systematic review of walking interventions in child and adolescent populations [52] and focus group discussions exploring the attitudes of adolescents towards physical activity [54]. The WISH intervention was based on the socioecological framework and informed by Self-Determination Theory (SDT) which provides a framework for understanding and enhancing the motivational mediators of behaviour change [44, 65, 66]. Within the design and delivery of the WISH intervention, the SDT domains of autonomy, competence and relatedness were addressed. For example, promoting knowledge and understanding of the health benefits of walking aimed to increase autonomous motivation for walking. The intervention design focused on short, brisk walks as opposed to longer bouts of activity to build pupil confidence in their ability to take part and maintain activity. In terms of relatedness, the inclusion of peer walk leaders and group-based intervention delivery was to support pupils to bond with their peers and provide social support. The present study was conducted according to the guidelines laid down in the Declaration of Helsinki, and all procedures were approved by Ulster University Research Ethics Committee (Ref: REC/19/0020) and written informed consent (parent/guardian) and assent (pupils) was obtained.

The full trial was conducted with 18 schools from Sept 2021-Nov 2022 (Phase 3). Initially, it was planned to conduct the study over a two-year period (Phase 1: 9 schools involved from Sept 2019-Oct 2020; Phase 2: 9 schools involved from Sept 2020-Oct 2021). However, due to the COVID-19 pandemic and associated closure of schools for face-to-face teaching, Phases 1 and 2 were suspended after mean intervention duration of 16 and 8 weeks respectively as it was not possible to run the intervention in full or complete data collection. Subsequently, the full trial was conducted from Sept 2021-Nov 2022 (Phase 3) and only data from Phase 3 will be reported in this manuscript. The trial registration was updated on 13th September 2021 to reflect the changes in the study planning [67].

### Sampling and participants

#### School recruitment

Using school enrolment data [68, 69], post-primary schools (mixed or single-sex) in the border region of Ireland and in NI were invited to participate provided they met the following criteria:

- NI: Schools with at least 80 girls across years 9–10 in counties Antrim, Armagh, Derry/Londonderry, Fermanagh and Tyrone.
- Ireland: Schools with an enrolment of > 240 girls in counties Cavan, Donegal, Leitrim, Louth, Monaghan and Sligo.

School enrolment data is presented differently in NI and Ireland. It was estimated that a total enrolment of 240 girls in Irish schools would equate to approximately 80 girls across 1st and 2nd year. Schools involved in Phase 2 of the WISH Study (September 2020-February 2021; suspended due to the COVID-19 pandemic) and allocated to the intervention group were not eligible due to potential contamination bias. Schools involved in Phase 1 were eligible to enrol for Phase 3 as the pupils who participated in Phase 1 had moved into Year 11/12 (NI) or 3rd/4th year (Ireland) at the point of recruitment for Phase 3. Schools that operated on multiple sites, where the older girls were on a different site to the younger girls were not eligible to participate.

Invitational letters accompanied with an expression of interest form were sent to School Principals and the Head of the PE Department of each eligible school. Follow-up contact was made via email and telephone. Sample size calculations have been documented previously [63] and were based on the WISH feasibility trial [58]. To detect a difference of 11.41 min total physical activity per day between groups, assuming a standard deviation (SD; pooled intervention and control group SD) of 25 min in total physical activity, a power of 80%, a significance of 0.05, an average cluster size of 20 children, an intra-class correlation of 0.03 [calculated from pilot data [58], and a coefficient of variation of 0.46, we needed a sample size of 15 schools increasing to 16 schools (8 per study arm) to allow for 10% cluster attrition. To allow for 20% loss to follow-up we recruited at least 24 children per cluster (at least 384 children in total). During the period of school recruitment, the COVID-19 pandemic was ongoing, schools and teachers were facing increased pressures so a further two schools were recruited to mitigate against possible increased attrition. To allow for 20% loss to follow-up we recruited at least 24 pupils per cluster. Participating schools were eligible to claim reimbursement for the use of their facilities and for teacher time (up to a maximum total of €400).

#### Participant recruitment

Following permission from the School Principal, girls in Year 9/10 (NI) and 1st/2nd year (Ireland) were invited by teaching staff to attend a recruitment presentation from the Trial Manager. The presentation informed pupils about the study, the randomisation process, and the intervention. Detailed information on participant recruitment has been provided previously [63]. For this project, ‘female’ was used as an adjective and ‘girl/girls’ as a noun (singular/plural, meaning an adolescent female human being [70]. The guidance to teaching staff was to invite girls in selected year groups to attend the recruitment presentation. No distinction was made between gender or sex and pupils were not asked to outline their sex to the research team. The recruitment strategy for this research study resulted in voluntary participation by those whose self-identified gender was girl and would be classified as ‘a girl’ within the NIH terms (i.e., adolescents assigned female (based on anatomy and physiology) at birth and whose self-identified gender is girl [71]) but data on gender/sex was not specifically collected. Decisions on who to invite to the recruitment presentations were made at a school-level and pupils who self-identified as ‘girl’ and voluntarily attended the recruitment presentation were eligible to participate. Therefore, it was the school, teacher(s), and voluntary participation of the child with associated parental consent that ensured those pupils that voluntarily identified as 'girls' (a key study inclusion criteria) were recruited for this research study. Study exclusion criteria were:

1. Boys were not eligible for inclusion, as it aimed to assess the effectiveness of a walking intervention targeted at adolescent girls only.
2. Pupils who are unable to walk or for whom walking is contraindicated were not eligible for inclusion as taking part in increased physical activity during the school day may not have been suitable for this population.

All eligible pupils who provided assent and written consent from their parent/guardian were invited to baseline data collection. Pupils not involved in the study as research participants but who wished to join the walks in intervention schools were welcome to do so. In intervention schools, female pupils aged 15–18 years were identified by staff and invited to train as walk leaders, lead the walks, and implement the WISH intervention within their school. Walk leaders and teachers were provided with a participant information sheet and asked to provide written informed consent. Participants (pupils, walk leaders and teachers) were free to withdraw from the study at any point without giving reason.

#### Randomisation

Schools were randomly allocated to control (*n* = 9) or intervention (*n* = 9) using the randomization.com webpage (http://www.randomization.com; accessed 24.11.2021). Schools were stratified by country and random allocation was performed by faculty staff blind to school identity and independent of the study team. Allocation was blinded (schools, pupils, and fieldwork research team) until baseline data collection had been completed. After baseline data collection schools were notified of their allocation. In schools allocated to the control group, pupils were asked to continue with their usual physical activity. Due to the nature of the intervention, it was not possible for schools, pupils, or the fieldwork research team to be blinded to allocation after baseline data collection.

#### Data collection

Data collection appointments were standardised across all schools and were completed at four time points, all appointments took place in schools and during school hours. Data were collected at baseline (T0: October–November 2021), mid-intervention (T1: February–March 2022; mean intervention duration at T1 = 10.3 ± 0.96 weeks), end of intervention (T2: May–June 2022; mean intervention duration at T2 = 19.9 ± 0.97 weeks), follow up (T3: October-December 2022; mean time since T0 data collection = 12.2 ± 0.3 months). Participants were asked to provide their date of birth to calculate age. For schools in Ireland, the Eircode (postcode) for each school was inputted to an interactive map (Pobal Interactive Map) and the ‘Pobal HP Index 2016’ and ‘Pobal HP Description 2016’ were recorded as a measure of socioeconomic status at school level [72]. The postcode for each school in NI was entered into the NI Multiple Deprivation Measures [73] and the deprivation score recorded.

#### Recruitment and retention to trial and intervention

Recruitment of schools, pupils and walk leaders was recorded at baseline. Retention of pupils and schools was recorded at T1, T2 and T3. Retention of walk leaders was recorded at T2. To minimise missing data, a second visit was arranged for those pupils that were absent on the day of the data collection visit. However, if the participant was absent at the second visit, further visits for that specific timepoint were not arranged.

##### Physical activity

The primary outcome measure was total physical activity (counts per minute, cpm) of pupils at T2 measured using the Actigraph accelerometer (Actigraph LLC, Florida) worn for seven consecutive days. The device was placed on an elastic waist band and pupils were asked to wear the accelerometer at all times, removing it only for bathing, water-based activities such as swimming and when asleep. Pupils were asked to wear the accelerometer on their right hip and to ensure that it was worn in the same position each day. To maximise accelerometer returns and minimise the amount of missing data, several strategies were implemented. During measurement periods, pupils were asked to keep a log of when they wore the accelerometer and took it off to encourage compliance with the wear-time protocol [74]. Each accelerometer was labelled with the contact information of the research team should a device be lost. Accelerometers were distributed face-to-face. Text messages were sent to the parent/guardian on day 7 to detail where and to whom the devices should be returned. Incentives were provided when pupils returned accelerometers. These incentives were chosen (water bottles, earphones, power-banks) and designed (hoodies) by the Youth Advisory Group (€17 total cost) [75]. All data were downloaded and analysed using ActiLife software (version 6.13.4; ActiGraph LLC, Florida, USA). Pupils were included in the analysis if they had ≥ 2 valid weekdays of data (500 min/day) [76]. All valid data was included in the analysis. Minutes of total physical activity (light, moderate and vigorous) per day were estimated using the Evenson cut-points [77]. A sampling epoch of 15 s was employed during data collection. Periods of ≥ 60 min of zero counts were categorised as ‘non-wear’ and removed. Accelerometer data was also used to calculate:

- Total physical activity (cpm) at T1 and T3
- Time spent in sedentary behaviour and light, moderate, MVPA and vigorous intensity physical activity [77] at T0, T1, T2 and T3
- Proportion of pupils meeting current physical activity recommendations of ≥ 60 min of MVPA per day [78] at T0, T1, T2 and T3
- Proportion of pupils achieving < 30 min, 30-60 min and > 60 min of MVPA per day at T0, T1, T2 and T3

As an additional exploratory analysis and based on the method of accelerometer data analysis within the feasibility study [58], a school-time filter, specific to each school’s start and finish time was applied. Pupils must have worn the device for a minimum of two full school days to be included in the school-time physical activity analysis. Data were analysed for time spent in sedentary behaviour and light, moderate, MVPA, and vigorous intensity physical activity across the school day at T0 versus T2.

##### Anthropometric measurements

The following anthropometric measurements were taken at T0, T2 and T3:

- Height (cm) and weight (kg) were measured to the nearest 0.1 cm and 0.1 kg, respectively, using a freestanding stadiometer (Leicester Height Measure), and digital scales (Seca 877) to calculate Body Mass Index (BMI). Measurements were completed by trained researchers. BMI was converted to an age-specific and gender-specific z-score using Stata v17 (StataCorp LLC, Texas, USA) [79–81].
- Waist and hip circumference were measured to the nearest 0.1 cm using an anatomical measuring tape and waist-to-hip ratio was calculated [82].

##### Questionnaires

Pupils were asked to complete a series of questionnaires at T0, T2 and T3 within the school premises on electronic devices (Apple iPad®) using Qualtrics (Provo, Utah, USA). The questionnaires were piloted and modified to ensure there was no ambiguity in the questions and to identify any potential problems the participant might experience. Questionnaires were estimated to take no longer than 30 min to complete. Data collected using the self-efficacy for physical activity and walking [58, 83], health-related quality of life [84], reasons for engaging in physical activity [85, 86] and friendship nominations questionnaires will be published in a separate process evaluation paper. During all data collection visits, questionnaires were completed before anthropometric measurements were taken. Self-reported measures for emotion regulation, body weight, appearance satisfaction, social media use and sleep quality will be reported in the present paper.

#### Emotion regulation

The Emotion Regulation Questionnaire for Children and Adolescents was used to assess cognitive reappraisal (changing the way one thinks about potentially emotion-eliciting events) and expressive suppression (changing the way one behaviourally responds to emotion-eliciting events) [87, 88]. This was a 10-item questionnaire and scores were calculated for cognitive reappraisal and expression suppression using a 7-point Likert scale, where higher scores on each subscale represented a greater use of that emotion regulation strategy. This instrument was evaluated with a sample of 827 participants (n469 (57%) female) aged between 10 and 18 years in Australia. Example items include “I control my feelings by not showing them” and in terms of construct validity, the comparative fit index was reported to be 0.942 and the root-mean-square error of approximation was 0.073 [87].

#### Body weight and appearance satisfaction

Using the questions adopted for the UK Millennium Cohort Study, body weight satisfaction was assessed using 3 questions previously used in an observational study of 10,904 14 year olds (n5496 girls; 50%) [89]: “Which of these do you think you are?” (underweight, about the right weight, slightly overweight, very overweight), “Have you ever exercised to lose weight or to avoid gaining weight?”, “Have you ever eaten less food, fewer calories or foods low in fat to lose weight or to avoid gaining weight?”. Responses were combined to generate a body satisfaction variable: satisfied or dissatisfied. Happiness with appearance was measured by asking, “On a scale of 1 to 7 where ‘1’ means completely happy and ‘7’ means not at all happy, how do you feel about the way you look?” Responses 1–6 were coded as ‘happy with appearance’ while a response of 7 was coded as ‘not happy with appearance’ [89].

#### Social media

There were three questions included in the questionnaire to assess social media usage (1) Do you use social media? (2) How often do you use social media? and (3) How many hours do you use social media on a typical day? Pupils were advised that, by social media, we meant Facebook, Twitter, Instagram, Snapchat, etc. Emotional investment in social media was assessed using the Social Media Use Integration Scale [90]. Similar to the methodology used by Woods and Scott [91], the term “social media” replaced “Facebook” in six of the questions. Items were rated on a 5-point Likert scale from “strongly disagree” to “strongly agree” and a higher overall score indicated a greater level of emotional investment in social media. Questions 1 to 6 assessed social integration and emotional connection to social media for example, “I get upset when I can’t log on to social media”, while questions 7 to 10 assessed integration into social routines [90] for example, “Using social media is part of my everyday routine”. This questionnaire was completed by 467 adolescents aged 11–17 years in Scotland and deemed to be reliable measure (Cronbach’s alpha = 0.78) [91].

#### Sleep quality

The Pittsburgh Sleep Index [92] is composed of 19 questions that assess 7 components (subjective sleep quality, sleep latency, sleep duration, sleep efficiency, sleep disturbance, use of sleep medication, and daytime dysfunction) and was used to measure sleep quality. Each question was scored 0 (no difficulty) to 3 (severe difficulty), and a global score was then calculated (0–21). A higher score indicated poorer sleep quality [92]. This measure is commonly used with adolescents and items include for example, “During the past month, how would you rate your sleep quality overall?”. In a validation study with 216 Spanish adolescents and young people (aged 14–24 years), moderate reliability was reported (Cronbach’s alpha = 0.72) [93], while when the instrument was used with a sample of Scottish adolescents, the Cronbach’s alpha value was 0.76 [91].

#### Intervention description

In intervention schools, school staff were asked to nominate female pupils aged 15–18 years (peer role models) with few existing extra-curricular commitments. These pupils were invited to train as walk leaders to lead younger pupils (aged 12–14 years) in 10–15-min walks before school and at break and lunch recess. In total, 149 walk leaders (mean age ± standard deviation (SD) 17 ± 0.8 years) were trained across the 9 intervention schools (mean number of walk leaders per school 17 ± 7). The intervention has been described in further detail previously [63]. In brief, the training programme for walk leaders was developed in consultation with the Physical Activity Co-Ordinator (Western Health and Social Care Trust) and was based on the *Walking For Health* Training (Public Health Agency & NI Health and Social Care Trusts). Training was theoretically underpinned by SDT and similar to the Bristol Girls Dance project [94], the training facilitator adopted an autonomy-supportive teaching style that strengthened walk leaders’ personal resources [63]. Training included an overview of the WISH Study, an understanding of health walks, the expectations of WISH walk leaders, knowledge of how to plan walks and keep walkers motivated. The training programme also included information on providing and encouraging social support among students to align with SDT. Walk leaders were provided with wrist worn heart rate monitors (Mi Band 3, Xiaomi, China) to guide the pace of the walk. Walk leaders were advised to check that their heart rate was approximately > 135 bpm (based on an estimated 70% maximal heart rate for a 16 year old) [95] and indicative of a brisk pace. The heart rate monitors were a feedback tool and used to provide reassurance to walk leaders that the walks were of sufficient intensity. The walk leaders were asked to monitor the pace of the walk, regularly remind pupils that they should be walking at a brisk pace and although there would have been variations in fitness and perceived effort among pupils, this was reflective of a walking group. For most pupils, if they were walking at the same pace as the walk leaders, they would have been walking at a brisk pace. Walk leaders were also trained to look out for other signs that they were walking at a brisk pace (breathing a little faster; feeling a little warmer; feel their heart beating a little faster; still able to hold a conversation) should they choose not to wear the heart rate monitor. Cue-cards were also provided to remind the walk leaders to ensure that the walking pace was brisk, and the walk leaders were asked to refer to these cards during the walks. The heart rate monitors were used only as a tool to guide the pace of the walk and were not used for data collection purposes. Walk leaders self-reported their physical activity, self-efficacy for physical activity and physical activity enjoyment pre- and post-intervention. These findings will be reported as part the process evaluation. All walks took place in school grounds, schools were asked to schedule up to 3 walks per day (before school, at break and at lunch recess). If it was not possible to schedule 3 walks per day, at a minimum, schools were requested to schedule 2 walks per day. Pupils could self-select the walks to attend and pupils were encouraged to participate in as many walks as possible each week. Two walk leaders accompanied each walk, one at the front and one at the back of the group. If possible, a third walk leader was present in the middle of the group. Walk leaders encouraged the younger pupils, set the pace of the walk, and ensured the safety of pupils. As part of the training and through regular communication, walk leaders were encouraged to decide the format of the walks for example, the use of music or games and routes taken. They could also choose to introduce themed walks (for example, Easter Treasure Hunt). The training also informed walk leaders of the support available for them in this role. Walk leaders were able to self-select the walks that they would like to lead, and the timetable was determined by the preferences of the walk leaders. Within a supportive training environment, walk leaders were provided with the skills and competence to run the walking programme within their school.

Following baseline measurements, the intervention was delivered for the whole school year. Allowing for holidays, exams, educational trips, and other school events intervention length varied across schools but was offered for a minimum of 18 and a maximum of 21 weeks (mean 19.9 ± 0.97 weeks). As part of the social support component of the intervention, walk leaders were invited to join a separate closed social media group/page (Facebook and Instagram) which included opportunities to share progress and useful information. Social support and encouragement were also provided to walk leaders via regular updates from the research team which included: strategies to address barriers to participation, recognising progress; links to websites and resources; and vignettes of support and advice from other walk leaders. The social media pages were only accessible to those in the intervention and were moderated by named members of the research team for data protection purposes.

A booster session was held in each intervention school. The aim of this session was to rejuvenate the programme after the Easter holidays, improve engagement and meet with walk leaders and the younger pupils together to discuss any potential issues. Each booster session took place 2–6 weeks (mean 4.1 ± 1.4 weeks) before the end of intervention data collection. Sessions lasted approximately 60 min and were led by the Trial Managers. Walk leaders and the younger pupils all attended the session where they received an update on study progress, the purpose of the intervention, incentives for taking part (walk leaders) or for taking part in data collection (younger pupils). All in attendance had the opportunity to provide verbal feedback on the programme and issues including implementation of the intervention, attendance at walks, walking timetables and walking routes were discussed. During this session, younger pupils from each school nominated a Walk Leader Champion to act as the main contact with the Research Team. The booster session ended with a group walk to emphasise again the importance of walking at a brisk pace.

#### Attendance at walks

The fidelity of each walk was assessed through a self-report checklist completed by walk leaders. This noted attendees, walk duration and location (indoor/outdoor).

##### Data analysis

Data were entered electronically on a secure file storage system and password protected. Data were anonymised by assigning a unique identification number to each pupil. All paper data entry forms were entered and then checked by another researcher. All data files were inspected for omissions and errors. Double data checking was completed for the primary (total physical activity) and secondary outcomes. All inconsistencies or implausible values were investigated and rectified. Missing and out-of-range data were checked against paper data collection forms to confirm values and correct them if necessary. The statistician (IML) who performed the data analyses was blinded to allocation throughout the study and data analysis did not occur until the Statistical Analysis Plan had been finalised, added to the Trial Registration [67] and sent to the External Member of the Trial Steering Committee (TSC).

The Statistical Analysis Plan has been published previously [67]. In brief, the main analysis for each outcome was undertaken on a modified ‘intention-to-treat’ (ITT) basis, i.e. all pupils with a recorded outcome at T0 and T2 were included in the analysis and were analysed according to the group to which they were allocated. A similar approach was used for the analysis of outcomes at T0 and T1 and at T0 and T3. Pupils were included in the analysis regardless of adherence to the physical activity intervention. The number of and reasons for loss to follow-up was reported, and the baseline characteristics of pupils included in the analysis to those excluded from the analysis was conducted. All quantitative analysis was conducted using R [96] and RStudio [97] version 2023.6.1.524 (release Mountain Hydrangea). Statistical tests and confidence intervals were two-sided. Between-group comparisons were calculated and presented with 95% confidence intervals wherever possible. The statistical significance level was set at the 5% level for the primary analysis. For secondary outcome analyses, since no statistical power calculations were undertaken for these assessments, these analyses were presented with point estimates and 95% confidence intervals but without *p*-values.

**Fig. 1:**
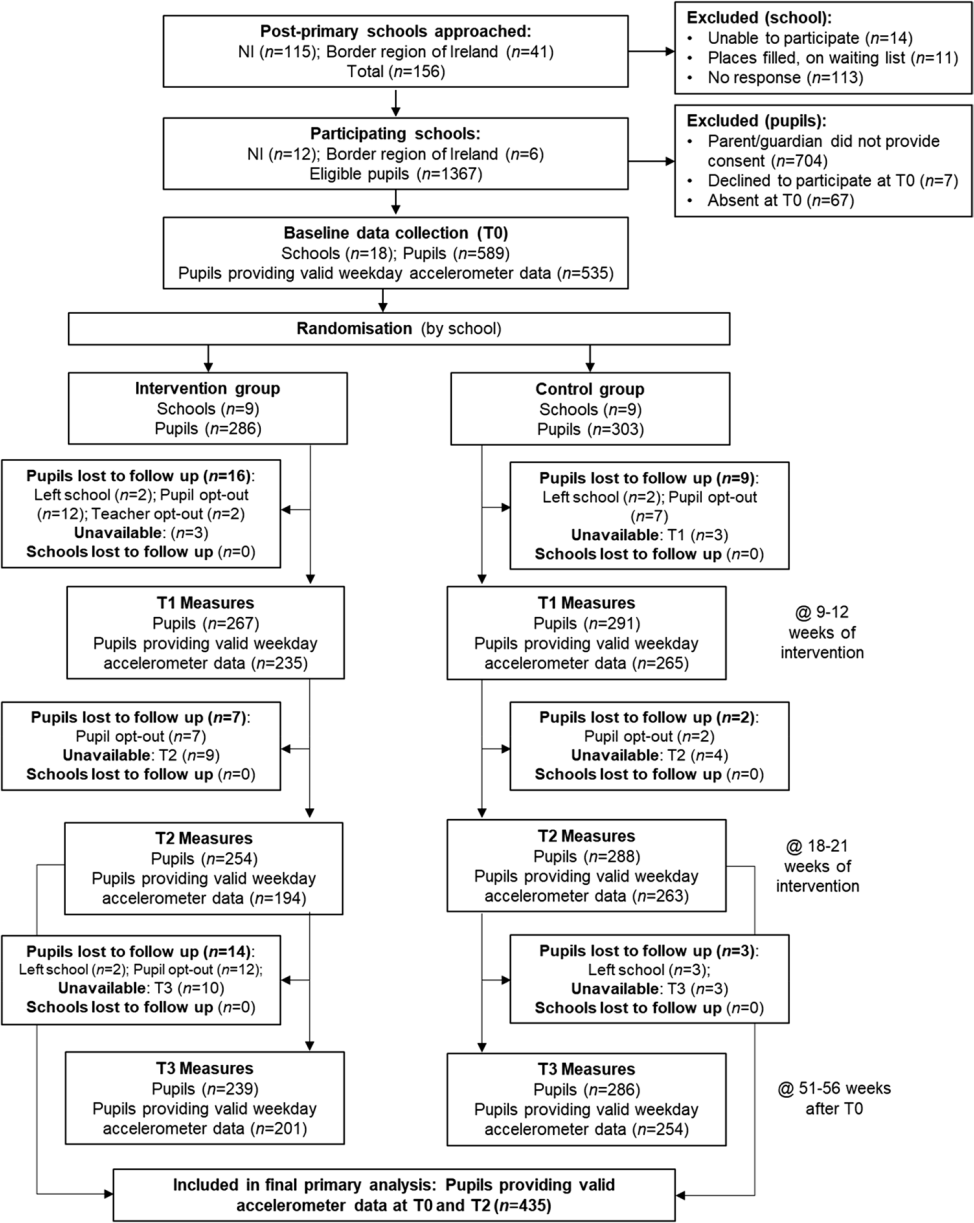
CONSORT Flow Diagram

All comparative analyses allowed for the clustered nature of the of the data and ensured correct confidence intervals and type I error rates were calculated. For each outcome, unless otherwise specified, the main analysis was the covariate-adjusted analysis, which adjusted for the child-level covariates (level 1): baseline value for the outcomes of interest [e.g., baseline total physical activity (cpm) in the primary analysis], age, and BMI z-score. Unadjusted between-group differences were presented for completeness. School and student recruitment and retention were presented as a CONSORT flow chart for schools by trial arm (Fig. 1). Recruitment, retention, and data provision were also summarised descriptively.

## Results

Eighteen schools were recruited with 589 pupils, of which 286 (48.6%) were in the nine schools allocated to the intervention arm (Fig. 1). Of the participating schools, 12 (*n* pupils = 385, 65%) were in NI and 6 (*n* pupils = 201, 35%) were in Ireland. The cluster size ranged from 25 to 51 pupils (mean 32.7 ± 6.6). Baseline characteristics of intervention (*n* = 286) and control (*n* = 303) pupils are shown in Table 1. Across all timepoints, less than 1% of pupils refused to have their weight measured but completed all other anthropometric measurements (height, waist and hip circumference). There were differences in the classification of deprivation levels between the two jurisdictions (Ireland: Pobal Interactive Map [72]; NI: Multiple Deprivation Measures [73]). Of the Irish schools (*n* = 6) involved, *n* = 3 (50%) were classified as disadvantaged and *n* = 3 (50%) were marginally below average in terms of deprivation based on the Pobal HP description [72]. Of the NI schools involved, *n* = 2 (16.7%) were in the top 20% of deprived areas [73].

**Table 1:** Characteristics of sample at baseline (T0)

Variable	Overall (*n* = 18 schools; *n* = 589 pupils)	Intervention (*n* = 9 schools; *n* = 286 pupils)	Control (*n* = 9 schools;* n* = 303 pupils)
Country, N (%):			
Ireland	204 (34.6%)	85 (29.7%)	119 (39.3%)
NI	385 (65.4%)	201 (70.3%)	184 (60.7%)
Age in years; median (IQR; range)	13 (1.0; 12–14)	13 (1.0; 12–14)	13 (1.0; 12–14)
Height (cm); mean (SD)	158 (7)	158 (6)	159 (7)
Weight (kg); median (IQR; range)	52 (14; 31–142)	52 (14; 32–127)	51 (14; 31–142)
BMI z-score; mean (SD)	0.65 (1.21)	0.66 (1.20)	0.64 (1.22)
BMI categories [79], N (%):			
Underweight	45 (7.6%)	20 (7.0%)	25 (8.3%)
Normal weight	321 (54.5%)	157 (54.9%)	164 (54.1%)
Overweight	131 (22.2%)	65 (22.7%)	66 (21.8%)
Obese	86 (14.6%)	41 (14.3%)	45 (14.9%)
Waist circumference (cm); median (IQR; range)	69 (13; 53–131)	69 (13; 53–121)	70 (12; 54–131)
Hip circumference (cm); median (IQR; range)	88 (11; 63–133)	88 (11; 63–133)	87 (11; 70–127)
Waist:Hip ratio; median (IQR; range)	0.8 (0.1; 0.7–1.1)	0.8 (0.1; 0.7–1.0)	0.8 (0.1; 0.7–1.1)

*NI* Northern Ireland, *IQR* Interquartile range, *SD* Standard deviation, *BMI* body mass index

### Accelerometer wear time

The median overall wear time was between 5 and 6 days at each time point (Supplementary Table 1). The number of pupils meeting the wear time criterion (at least 2 days) was highest at baseline (T0) (*n* = 535, 91%), but this decreased to 84% (*n* = 457) and 87% (*n* = 455) by T2 and T3, respectively. The control group had a higher proportion of pupils with sufficient wear time and a lower proportion of missing data than the intervention group at each time point (Supplementary Table 1). Age, height, weight, BMI z-scores, waist circumference, hip circumference, and waist-to-hip ratio of pupils who met the minimum wear time criteria were similar to those who did not meet this criterion at T0, T2, and T3 (Supplementary Table 2).

### Total physical activity

Age, BMI z-scores, and T0 total physical activity levels were similar between those providing T2 physical activity data and those with missing data at this timepoint (i.e. missing either due to insufficient wear time, pupil withdrawal from the study, or any other reason for not providing physical activity data; Supplementary Table 3). However, missing T2 physical activity data, was related to group allocation and school (Supplementary Table 3). We found higher proportions of missing physical activity at T2 in the intervention versus control group (missing: 32% vs. 13%) and across certain schools (min–max % missing: 0–53%). Therefore, it was not assumed that data were missing at random, and consequently, no imputation of missing values was performed. There were *n* = 435 pupils who had physical activity data at both T0 and T2 (*n* = 154 pupils missing/insufficient data at T0 or T2), with *n* = 179 in the intervention group (*n* = 107 pupils missing/insufficient data at T0 or T2) and *n* = 256 in the control group (*n* = 47 pupils missing/insufficient data at T0 or T2). The total and number of pupils per group providing physical activity data for other timepoints are presented in Table 2.

**Table 2:** Physical activity outcomes

**Outcome**		**Intervention**		**Control**		**Between-group differences (1–0)**^d^		
	**Time**	**N (%)**	**Median (IQR)**	**N (%)**	**Median (IQR)**	**EMM**^a^	**95% CI**	**ICC**
Total physical activity (cpm)	T0	248 (87)	695 (243)	287 (95)	673 (217)			
	T1	235 (82)	682 (261)	265 (88)	681 (273)	-22.5	-67.4 to 22.3	0.07
	T2^b^	194 (68)	683 (299)	263 (87)	675 (268)	-33.5	-88.1 to 21.2	0.09
	T3	201 (70)	608 (274)	254 (84)	626 (249)	7.5^c^	-28.5 to 43.6	0.05
MVPA (min/d)	T0	248 (87)	36.1 (23.0)	287 (95)	35.3 (19.8)			
	T1	235 (82)	36.6 (25.6)	265 (88)	38.2 (22.8)	-1.1	-5.6 to 3.4	0.07
	T2^b^	194 (68)	38.6 (29.5)	263 (87)	36.8 (22.3)	-1.5	-6.6 to 3.6	0.08
	T3	201 (70)	33.3 (25.1)	254 (84)	32.8 (22.2)	0.1	-6.3 to 6.5	0.17
Vigorous physical activity (min/d)	T0	248 (87)	8.4 (9.6)	287 (95)	8.5 (9.1)			
	T1	235 (82)	9.1 (11.9)	265 (88)	9.1 (11.3)	-0.8	-2.9 to 1.3	0.06
	T2^b^	194 (68)	9.1 (12.0)	263 (87)	8.8 (10.0)	-0.6	-2.4 to 1.2	0.02
	T3	201 (70)	6.8 (9.7)	254 (84)	7.4 (11.6)	-0.1	-2.8 to 2.7	0.12
Moderate physical activity (min/d)	T0	248 (87)	27.7 (15.1)	287 (95)	25.9 (12.5)			
	T1	235 (82)	27.6 (14.8)	265 (88)	25.9 (15.0)	-0.2	-3.2 to 2.8	0.08
	T2^b^	194 (68)	28.1 (16.4)	263 (87)	26.5 (12.3)	-0.8	-4.6 to 3.0	0.11
	T3	201 (70)	24.5 (14.4)	254 (84)	24.6 (13.7)	0.1	-3.8 to 4.1	0.14
Light physical activity (min/d)	T0	248 (87)	191 (55)	287 (95)	187 (60)			
	T1	235 (82)	176 (54)	265 (88)	185 (59)	-8.6	-16.9 to -0.3	0.02
	T2^b^	194 (68)	174 (60)	263 (87)	185 (66)	-8.6	-21.6 to 4.4	0.07
	T3	201 (70)	167 (54)	254 (84)	171 (54)	1.6	-10.3 to 13.5	0.09
Sedentary behaviour (min/day)	T0	248 (87)	539 (81)	287 (95)	544 (101)			
	T1	235 (82)	534 (91)	265 (88)	542 (112)	-3.3^c^	-10.6 to 17.2	0.02
	T2^b^	194 (68)	548 (104)	263 (87)	547 (119)	1.4	-19.4 to 22.1	0.05
	T3	201 (70)	545 (96)	254 (84)	554 (89)	-5.5	-23.9 to 13.0	0.05

*IQR* Interquartile range, *CI* Confidence interval, *ICC* Intraclass Correlation Coefficient, *EMM* Estimated marginal means, *MVPA* Moderate-to-vigorous physical activity, *T0* Baseline, *T1* Mid-intervention, *T2* End of intervention, *T3* 12-month follow-up

^a^Estimated marginal means at each timepoint were adjusted for baseline measure of the outcome (e.g., total physical activity in cpm) at baseline, BMI z-score, and baseline age

^b^Primary outcome

^c^Robust linear mixed model analysis estimates reported

^d^Complete case analysis for T0-T2: n435 pupils overall (n179 intervention group; n256 control group); T0-T1: n471 pupils overall (n212 intervention group; n259 control group); T0-T3: n439 pupils overall (n190 intervention group; n249 control group)

At baseline, pupils in both groups engaged in a median (IQR; min–max) of 683 (229; 302–1849) cpm of physical activity. The mean post-intervention total physical activty for the intervention group was 676 cpm and 710 cpm in the control group (Table 2). Post-intervention total physical activity did not statistically differ between groups when adjusted for age, body mass index z-scores and baseline physical activity (mean difference, -33.5, 95% CI = -21.2 to 88.1; *p* = 0.213, Supplementary Table 4a). Similar findings were provided for a model adjusted only for baseline total physical activity (unadjusted analysis, see Supplementary Table 4b). Although the control group had statistically (albeit only slightly) higher light physical activity versus the intervention group at T1, there were no between-group differences for the other secondary physical activity outcomes and sedentary behaviour at any timepoint providing an overall indication that the intervention did not have a beneficial effect on levels of physical activity (Table 2).

At baseline, some 12% (*n* = 66) of pupils were achieving the recommended 60 min of MVPA per day [6], (15% of intervention pupils and 10% of control pupils respectively), Supplementary Table 5.

### School-time physical activity

At baseline, the median (IQR) total physical activity was 635 (238) cpm for the intervention group and 614 (208) cpm for the control group. At the end of the intervention, total physical activity decreased to median (IQR) 572 (302) cpm in the intervention group and 532 (208) cpm in the control group. As outlined in Table 3, there were no differences between the groups for time spent in sedentary behaviour, total, light, moderate, or vigorous intensity physical activity across the school day at end of intervention.

**Table 3:** School-time physical activity

**Outcome**		**Intervention**		**Control**		**Between-group differences (1–0)**		
	**Time**	**N (%)**	**Median (IQR)**	**N (%)**	**Median (IQR)**	**EMM**	**95% CI**	**ICC**
Total physical activity (cpm)	T0	248 (87%)	635 (238)	273 (90%)	614 (208)			
	T2	189 (66%)	572 (302)	260 (86%)	532 (208)	23.7	-79.4 to 126.8	0.34
MVPA (min/d)	T0	248 (87%)	19 (13)	273 (90%)	18 (10)			
	T2	189 (66%)	17 (13)	260 (86%)	15 (10)	1.7	-3.1 to 6.5	0.32
Vigorous physical activity (min/d)	T0	248 (87%)	3 (4)	273 (90%)	3 (4)			
	T2	189 (66%)	3 (5)	260 (86%)	2 (3)	0.9	-0.7 to 2.5	0.15
Moderate physical activity (min/d)	T0	248 (87%)	15 (9)	273 (90%)	14 (7)			
	T2	189 (66%)	14 (8)	260 (86%)	12 (7)	0.9	-2.6 to 4.3	0.34
Light physical activity (min/d)	T0	248 (87%)	79 (30)	273 (90%)	79 (28)			
	T2	189 (66%)	71 (26)	260 (86%)	71 (35)	0.1	-10.8 to 11.1	0.25
Sedentary behaviour (min/day)	T0	248 (87%)	289 (34)	273 (90%)	290 (35)			
	T2	189 (66%)	296 (41)	260 (86%)	300 (35)	-1.1	-16.7 to 14.5	0.32

*IQR* Interquartile range, *CI* Confidence interval, *ICC* Intraclass Correlation Coefficient, *EMM* Estimated marginal means, *MVPA* Moderate-to-vigorous physical activity, *T0* Baseline, *T2* End of intervention

### Self-reported measures of health and wellbeing

Across the four questionnaires included in this analysis, the proportion of missing data was low and ranged from 0 to 11.5% at all timepoints. Imputation for missing data was not undertaken as data were not missing at random. There were differences in the proportion of missing data between groups (intervention and control) and between schools.

### Emotion regulation

At baseline, the median (IQR) score for cognitive appraisal was 28 (8) and 27 (8) for the intervention and control groups, respectively. The median (IQR) score for expressive suppression was 15 (6) in both the intervention and control groups and no between-group differences were observed for cognitive reappraisal or expressive suppression at any timepoint.

### Body weight & appearance satisfaction

Most pupils responded that they were happy with their appearance (95%) but dissatisfied (74%) with their bodyweight at baseline and this was similar between intervention and control groups (Table 4). Adjusting for baseline age, BMI z-score, and satisfaction with bodyweight, there were between-groups differences observed in happiness with appearance at T2 and T3, and satisfaction with bodyweight at T2. The odds of pupils in the intervention group being satisfied with their bodyweight at T3 was 54% (95% CI = 22% to 73%) lower compared with the odds for those in the control group (Table 4). In addition, for every 1-unit increase in BMI the odds of being satisfied with bodyweight decreased by 47% (95% CI = 33% to 57%).

**Table 4:** Bodyweight satisfaction and happiness with appearance

	**Overall**	**Intervention**	**Control**	**Difference**
	**N (%)**	**N (%)**	**N (%)**	**OR (95% CI)**
**Happiness with appearance** [89]				
Happy with appearance, T0:				
• Unhappy	32 (5%)	18 (6%)	14 (5%)	
• Happy	557 (95%)	268 (94%)	289 (95%)	
Happy with appearance, T2:				
• Unhappy	43 (8%)	17 (7%)	26 (9%)	0.66
• Happy	499 (92%)	237 (93%)	262 (91%)	(0.26, 1.70)
Happy with appearance, T3:				
• Unhappy	29 (6%)	15 (6%)	14 (5%)	1.29
• Happy	491 (94%)	223 (94%)	268 (95%)	(0.56, 2.99)
**Satisfied with bodyweight** [89]				
Satisfied with bodyweight, T0:				
• Dissatisfied	433 (74%)	215 (75%)	218 (72%)	
• Satisfied	156 (26%)	71 (25%)	85 (28%)	
Satisfied with bodyweight, T2:				
• Dissatisfied	413 (76%)	194 (76%)	219 (76%)	0.96
• Satisfied	129 (24%)	60 (24%)	69 (24%)	(0.57, 1.60)
Satisfied with bodyweight, T3:				
• Dissatisfied	398 (77%)	169 (71%)	229 (81%)	0.46
• Satisfied	122 (23%)	69 (29%)	53 (19%)	(0.27, 0.78)

*CI* Confidence Interval, *OR* Odds ratio, *T0* Baseline, *T2* End of intervention, *T3* 12-month follow-up

### Social media

At baseline, most participants (*n* = 524, 89%) used social media daily (89% in both intervention and control groups). At the end of the intervention, *n* = 506 (93%) of participants used social media 2–3 times per week (93% intervention vs 94% control group). At 12-month follow-up, there was a further reduction in the frequency of social media use with the majority *n* = 494 (94%) of participants stating that they used social media once per week (94% in both the intervention and control groups). In terms of time spent on social media, only 6.5% of participants said they spent less than one hour per day on social media at baseline. 37.4% spent 1–3 h per day, 39% spent 3–6 h per day and 13.4% spent ≥ 6 h per day on social media with no between-group differences in emotional investment in social media (Table 5). There were no differences in physical activity (cpm) between the intervention and control groups at T2 when adjusting for baseline physical activity and time spent on social media.

**Table 5:** Emotional investment in social media

**Outcome**		**Intervention**		**Control**		**Between-group differences (1–0)**		
	**Time**	**N (%)**	**Median (IQR)**	**N (%)**	**Median (IQR)**	**EMM**^a^	**Unadjusted 95% CI**	**ICC**
Total social media use integration [90]	T0	271 (95)	39.0 (15.0)	282 (93)	39.0 (15.0)			
	T2	241 (84)	41.0 (14.0)	276 (91)	41.0 (14.0)	-1.47	-3.15 to 0.21	0.00
	T3	224 (78)	42.0 (14.0)	275 (91)	42.0 (14.0)	0.10	-2.83 to 3.04	0.08
Integration into social routines [90]	T0	271 (95)	18.0 (6.0)	282 (93)	18.0 (6.0)			
	T2	241 (84)	20.0 (6.0)	276 (91)	20.0 (7.0)	-0.34	-1.10 to 0.41	0.01
	T3	224 (78)	20.0 (5.0)	275 (91)	20.0 (5.0)	0.16	-1.03 to 1.34	0.08
Social Integration Emotional Connection [90]	T0	271 (95)	20.0 (10.0)	282 (93)	20.0 (11.0)			
	T2	241 (84)	22.0 (10.0)	276 (91)	23.0 (11.0)	-1.16	-2.34 to 0.02	0.00
	T3	224 (78)	23.0 (9.3)	275 (91)	24.0 (9.0)	-0.10	-1.96 to 1.77	0.06

*IQR* Interquartile range, *EMM* Estimated marginal means, *ICC* Intra-class correlation coefficient, *T0* Baseline, *T1* Mid-intervention, *T2* End of intervention, *T3* 12-month follow-up

^a^Estimated marginal means at each timepoint were adjusted for baseline measure of the outcome (e.g., total physical activity in cpm) and baseline BMI z-score, and baseline age

### Sleep quality

At baseline, the median (IQR) score for sleep quality was 6 (3) and 4 (4) for the intervention and control groups, respectively. There were no between-group differences for sleep quality at any timepoint.

### Attendance at walks

Fidelity checklists recorded a total of 371 walks across the intervention. There was variation in the number of walks recorded per school (range 2–104). Schools were asked to schedule up to 3 walks per day (before school, at break and at lunch recess). If it was not possible to schedule 3 walks per day, at a minimum, schools were requested to schedule 2 walks per day, however based on data collected from the fidelity checklists, the mean number of walks that took place per school was 41.2 ± 34.4 which equated to 2.1 walks per school per week of the intervention. In terms of the size of the walking groups, the mean number of pupils per intervention school was 32 ± 6, while the mean attendance per walk was 6 ± 5 (range 0 – 30 pupils). In total, *n* = 74 (25.9%) of intervention pupils did not participate in any walks over the intervention. The mean number of walks attended per pupil was 8.1 ± 14.2. The mixed-methods process evaluation followed the RE-AIM Framework and explored factors that impacted the delivery and implementation of the intervention. To allow adequate space to fully discuss the process evaluation, results will be reported elsewhere.

## Discussion

The limited effectiveness of previous physical activity interventions among adolescent girls has led to calls for more novel school-based approaches and improvements to the quality of physical activity opportunities offered across the school day [98, 99]. The WISH intervention was considered novel as the effectiveness of a peer-led walking intervention among adolescent populations was untested in a fully powered trial. Despite the changes observed during the pilot study [58], the data presented in this paper suggests that the WISH intervention did not increase device measured physical activity or reduce sedentary behaviour when delivered across an academic school year. Given the reported associations between physical activity and mental wellbeing [100], sleep [101], body image [102], and social media [103], a number of questionnaires were used to assess any changes as a result of the WISH intervention. Considering that physical activity behaviour did not change, it was therefore not unexpected that sleep quality, emotional investment in social media, emotion regulation and body weight & appearance satisfaction did not differ between the control and intervention groups.

Overall, the findings reported in this study were surprising as the feasibility study conducted in six schools (2 intervention and 4 control, *n* = 199 pupils) showed that the 12 week walking intervention increased physical activity across the school day by 8.27 min/day compared to a decrease of 2.14 min/day in the control group [58]. The relatively short intervention period (12 weeks) and small sample size of the feasibility study indicated that a larger evaluation was necessary to ensure that the findings reported in the feasibility study could be maintained when ‘scaled-up’. Feasibility studies are a key stage in the development of almost all large-scale, efficacy/effectiveness intervention trials, however it is acknowledged that generalisability biases can impact the success of scaling-up from feasibility to definitive trial in interventions [104].

It is important to acknowledge that the intervention was conducted against the background of the COVID-19 pandemic. The overall study period was from September 2021 to October 2022, schools had reopened after COVID-19 lockdowns however, many of the school-based measures introduced to reduce infection rates remained in place [105]. The intervention was planned for the full school year, however due to the COVID-19 pandemic, it was no longer possible to conduct the study in two phases (over two years) and all baseline data collection had to be completed before schools were randomised to control or intervention. This meant that intervention start date ranged from late November to early December 2021. In addition, although the intervention commenced in all schools before the Christmas holidays, on the return to school, teachers reported an increase in pupil and walk leader absences due to COVID-19 which had implications for restarting the programme in early 2021.

Although, this was a robust and well-planned intervention, changes to school environments may have affected the implementation of the intervention. For example, many schools used a staggered break and lunch time schedule meaning that the walk leaders may have had a different break and lunch time to the intervention pupils affecting the involvement of walk leaders in the programme. Some schools continued to place students in class/year group bubbles to ensure social distancing and in some schools the sports hall was used as additional teaching space which had implications for being able to offer an indoor walking route in the event of adverse weather. Mask wearing was compulsory in NI schools until 21st March 2022 and in schools in Ireland until 28th February 2022 [106, 107]. Research suggests that face masks have little effect in low to moderate intensity activities as the physiological demands are low [108], however, it is unknown if face masks were a barrier to participating in the walks among this cohort. Full details on the intervention delivery, implementation and factors that affected participation, motivation, and enjoyment of the intervention will be reported in a separate process evaluation paper.

Pupil and staff absences were high in this academic year. For example, within the six NI intervention schools, the proportion of pupils absent from school was highest in 2021/22 (11.2%), compared with 2020/21 (6.6%) and 2019/20 (8.1%) [109]. In addition, for the 2021/22 academic year, a total of 34,972 calendar days were lost as a result of staff absences due to COVID-19 in NI post-primary schools [110]. For pupils, the return to school in September 2021, was reported as being a particularly difficult transition and following the easing of restrictions, children reported emotional overwhelm and physical fatigue leading to the avoidance of physical activity [111].

Changing physical activity levels among adolescent girls through school-based interventions is challenging, as reported in several systematic reviews and meta-analysis [31, 112, 113]. Consistent with the results of several recent robust and well-designed studies, this study adds to the evidence that school-based physical activity interventions have had limited impact on the physical activity levels of adolescent girls. The PLAN-A intervention was a peer-led physical activity programme which ran for 5–6 months and was implemented in 10 post-primary schools, the intervention did not change accelerometer-measured weekday MVPA or associated secondary outcomes [20]. The Girls Active project which provided a support framework for 10 intervention schools to review and change their physical activity policies did not change accelerometer-measured MVPA at 14 month follow-up [114].

In theory, the WISH intervention held considerable promise, it was a low-cost intervention that was enjoyed by pupils and walk leaders and viewed positively by teachers. However, issues around the implementation, school environment and attendance at walks meant that accelerometer-measured physical activity and associated secondary outcomes were unchanged in this c-RCT. A number of strategies were implemented to encourage attendance at the walks; the implementation of the intervention was informed by Patient and Public Involvement (PPI) [75], reward cards were used for pupils to self-monitor their attendance [63] and a booster session was held to rejuvenate the programme. Despite these efforts, a small number of walks were initiated, and implementation of the intervention varied across schools. A criticism of previous school-based interventions is the lack of focus on the school environment [115] and there have been recent calls for researchers to focus “on creating the best intervention approach for each setting for the agreed outcome of interest, and then seek an evaluation design that facilitates the assessment of the efficacy of that approach” [115]. Context, which incorporates the school setting, ethos, staff, facilities, and sociodemographic factors is key to effective school-based physical activity interventions but has rarely been considered in intervention design [115]. Reflecting on the WISH intervention, particularly post-pandemic, leads the authors to conclude that there is a need to consider a context-specific approach for future school-based physical activity interventions as an alternative to the cluster RCT study design to evaluate such interventions [115].

### Strengths and limitations

The main strength of this study was the robust methodology employed. The WISH Study methods, including the statistical analysis plan were prospectively registered. The intervention was aligned to the MRC Framework for complex interventions [64] and followed a promising feasibility study [58]. The feasibility study was informed by qualitative research with adolescents [54]. The design and implementation of the definitive trial was guided by the Youth Advisory Group (YAG) [75]. The conducted study adhered to the previously published study protocol [63], the trial was registered prospectively and an independent External Member of the TSC was appointed. Sample size and cluster size requirements were met for each timepoint. Physical activity was measured using accelerometers and the study was fully powered to detect differences in total physical activity. A largely inactive population was recruited and baseline physical activity levels were consistent with previously reported physical activity levels in this population group [26, 29, 116]. Data were not analysed until the statistical analysis plan had been sent to the external member of the TSC and uploaded to the trial registration. Across four data collection timepoints, *n* = 2213 accelerometers were issued to participants. Only *n* = 3 (0.14%) devices were unreturned. Pupils were engaged with data collection and at all timepoints > 84% of enrolled pupils met wear-time criteria.

A limitation of this study was the use of fidelity checklists to monitor adherence to the intervention and attendance at walks. Despite frequent reminders and options to complete the checklist online or return a hard copy form, walk leaders reported forgetting to complete the forms and, in some cases, forms were completed poorly. As a result, it is not possible to determine with certainty how many walks occurred over the course of the intervention. Some walks may have taken place but due to walk leader absences were undocumented. Although the planned intervention duration was to be a minimum of 20 weeks, this was more challenging in schools in Ireland as their academic year ended 4–6 weeks before schools in NI; for two of these schools, the intervention duration was 18 weeks (excluding school closures). Lastly, the accelerometer data analysis outlined in the present paper was based on a robust and well-established method that uses cut-points to estimate time spent in different movement intensities [77]. However, in recent years, the methods used to analyse accelerometer data have evolved and there is growing consensus that raw acceleration data (average acceleration and intensity gradient) metrics should be used to fully describe physical activity profiles [117, 118]. Although it is beyond the scope of this paper, future research should consider this type of analysis to provide further insight and increase the comparability of accelerometer data.

There were several deviations from our original preregistered protocol and statistical plan. In our original statistical plan we had intended to adjust the false discovery rate using the Benjamini–Hochberg Procedure, however, we chose not to do this in keeping with the recommendations for multi-level models by Gelman and colleagues [119]. We also did not adjust for deprivation due to the differences in these measures across Ireland and NI. The booster sessions held in intervention schools to rejuvenate the programme after the Easter holidays were not part of our published study protocol but were considered necessary based on pupil engagement with the intervention.

## Conclusions

‘Scaling up’ physical activity interventions is challenging and despite a promising feasibility study, this definitive trial has demonstrated that in this context, the WISH intervention did not increase levels of physical activity or associated secondary outcomes among adolescent girls. In theory, the WISH intervention held considerable promise, it was a low-cost intervention that was enjoyed by pupils and walk leaders and viewed positively by teachers. However, school environments have changed due to the COVID-19 pandemic and there were issues around the implementation of the intervention. For future school-based physical activity interventions, there is a need to consider a context-specific approach.

### Supplementary Information

**Additional file 1: Supplementary Table 1.** Number (%) of pupils with sufficient wear time across groups and timepoints. **Supplementary Table 2.** Characteristics of pupils with sufficient vs insufficient wear time. **Supplementary Table 3.** Baseline (T0) characteristics of pupils who were missing physical activity data at baseline or end of intervention. **Supplementary Table 4.** a: Linear mixed model for the effect of intervention (group) on post-intervention (T2) physical activity (counts per minute, cpm). Adjusted for baseline age, BMI z-scores, and baseline total physical activity. **Supplementary Table 4. **b: Adjusted (left; adjusting for baseline age, BMI z-scores, and baseline total physical activity) and unadjusted (right; adjusted only for baseline total physical activity) linear mixed model for the effect of intervention (group) on post-intervention (T2) physical activity (counts per minute, cpm). **Supplementary Table 5.** The number (%) of pupils meeting recommended physical activity guidelines across groups and timepoints.

**Additional file 2.**

## Data Availability

The code created and datasets analysed for the current study will be available on the Open Science Framework (https://osf.io/).

## Abbreviations

BMI: Body mass index
CI: Confidence interval
cpm: Counts per minute
c-RCT: Cluster randomised controlled trial
EMM: Estimated marginal means
ICC: Intraclass Correlation Coefficient
IQR: Interquartile range
MRC: Medical Research Council
MVPA: Moderate-vigorous physical activity
NI: Northern Ireland
PE: Physical Education
SD: Standard deviation
SDT: Self-Determination Theory
TSC: Trial Steering Committee
WISH: Walking In ScHools Study
WHO: World Health Organisation
YAG: Youth Advisory Group
