# Supplementary material for: Effectiveness of the Walking in ScHools (WISH) Study, a peer-led walking intervention for adolescent girls: results of a cluster randomised controlled trial
Source: Int J Behav Nutr Phys Act. 2024 Feb 19;21:19. doi: 10.1186/s12966-024-01563-0 (PMC10877798; doi:10.1186/s12966-024-01563-0)
Supplement: Supplementary file 1 — Additional file 1: Supplementary Table 1. Number (%) of pupils with sufficient wear time across groups and timepoints. Supplementary Table 2. Characteristics of pupils with sufficient vs insufficient wear time. Supplementary Table 3. Baseline (T0) characteristics of pupils who were missing physical activity data at baseline or end of intervention. Supplementary Table 4. a: Linear mixed model for the effect of intervention (group) on post-intervention (T2) physical activity (counts per minute, cpm). Adjusted for baseline age, BMI z-scores, and baseline total physical activity. Supplementary Table 4. b: Adjusted (left; adjusting for baseline age, BMI z-scores, and baseline total physical activity) and unadjusted (right; adjusted only for baseline total physical activity) linear mixed model for the effect of intervention (group) on post-intervention (T2) physical activity (counts per minute, cpm). Supplementary Table 5. The number (%) of pupils meeting recommended physical activity guidelines across groups and timepoints. [file 12966_2024_1563_MOESM1_ESM.docx]

**Supplementary Table 1:** Number (%) of pupils with sufficient wear time across groups and timepoints

|  | **Overall (*n*=589)** | **Intervention (*n*=286)** | **Control (*n*=303)** |
| --- | --- | --- | --- |
| Median (IQR; min-max) total days wear time^a^: | | | |
| T0, baseline | 6 (3; 0-7) | 5 (3; 0-7) | 6 (2; 0-7) |
| T1, mid-intervention | 5 (3; 0-7) | 5 (2; 0-7) | 6 (3; 0-7) |
| T2, end of intervention | 5 (3; 0-7) | 4 (4; 0-7) | 5 (3; 0-7) |
| T3, 12-month follow-up | 5 (3; 0-7) | 5 (3; 0-7) | 5 (2; 0-7) |
| N (%) wear time ≥2 days at T0, baseline: | | | |
| Yes | 535 (91%) | 248 (87%) | 287 (95%) |
| No | 54 (9%) | 38 (13%) | 16 (5%) |
| Missing^b^ | 0 (0%) | 0 (0%) | 0 (0%) |
| N (%) wear time ≥2 days at T1, mid-intervention: | | | |
| Yes | 500 (85%) | 235 (82%) | 265 (87%) |
| No | 58 (10%) | 32 (11%) | 26 (9%) |
| Missing^b^ | 31 (5%) | 19 (7%) | 12 (4%) |
| N (%) wear time ≥ 2days at T2, end of intervention: | | | |
| Yes | 457 (78%) | 194 (68%) | 263 (87%) |
| No | 81 (14%) | 57 (20%) | 24 (8%) |
| Missing^b^ | 51 (9%) | 35 (12%) | 16 (5%) |
| N (%) wear time ≥ 2days at T3, 12-month follow-up: | | | |
| Yes | 455 (77%) | 201 (70%) | 254 (84%) |
| No | 69 (12%) | 37 (13%) | 32 (11%) |
| Missing^b^ | 65 (11%) | 48 (17%) | 17 (5%) |

IQR Interquartile Range, T0 Baseline, T1 Mid-intervention, T2 End of intervention, T3 12-month follow-up

^a^Valid wear time criteria defined as ≥2 valid weekdays of accelerometer data (500mins/day)

^b^Includes participants that withdrew from the study, were unavailable at that time point or did not return their accelerometer

**Supplementary Table 2:** Characteristics of pupils with sufficient vs insufficient wear time

|  | **Sufficient wear time**   (N: T0=533; T2=457; T3=455) | **Insufficient wear time**  (N: T0=54; T2=81; T3=69) |
| --- | --- | --- |
| Age at T0, N (%): |  |  |
| - 12 years | 228 (43%) | 16 (30%) |
| - 13 years | 250 (47%) | 27 (50%) |
| - 14 years | 55 (10%) | 11 (20%) |
| Age at T2, N (%): |  |  |
| - 12 years | 29 (6%) | 4 (5%) |
| - 13 years | 290 (64%) | 49 (61%) |
| - 14 years | 131 (29%) | 28 (35%) |
| - 15 years | 4 (1%) | 0 (0%) |
| Age at T3, N (%): |  |  |
| - 12 years | 0 (0%) | 0 (0%) |
| - 13 years | 176 (40%) | 29 (45%) |
| - 14 years | 207 (48%) | 26 (40%) |
| - 15 years | 50 (12%) | 10 (15%) |
| BMI categories at T0, N (%): |  |  |
| - Underweight | 43 (8%) | 2 (4%) |
| - Normal weight | 293 (55%) | 28 (54%) |
| - Overweight | 121 (23%) | 10 (19%) |
| - Obese | 74 (14%) | 12 (23%) |
| BMI categories at T2, N (%): |  |  |
| - Underweight | 32 (7%) | 8 (10%) |
| - Normal weight | 256 (57%) | 39 (48%) |
| - Overweight | 97 (22%) | 19 (24%) |
| - Obese | 65 (12%) | 15 (19%) |
| BMI categories at T3, N (%): |  |  |
| - Underweight | 29 (6%) | 6 (9%) |
| - Normal weight | 268 (59%) | 37 (55%) |
| - Overweight | 88 (20%) | 12 (18%) |
| - Obese | 66 (15%) | 12 (18%) |
|  |  |  |
| Median (IQR) BMI z-score at: |  |  |
| - T0 | 0.59 (1.62) | 0.82 (1.84) |
| - T2 | 0.56 (1.52) | 0.86 (1.63) |
| - T3 | 0.53 (1.54) | 0.64 (1.32) |
| Median (IQR) WC at: |  |  |
| - T0 | 69.4 (12.6) | 69.9 (13.9) |
| - T2 | 69.4 (13.5) | 70.0 (12.9) |
| - T3 | 71.7 (12.3) | 72.2 (10.4) |
| Median (IQR) HC at: |  |  |
| - T0 | 87.5 (11.3) | 86.9 (10.3) |
| - T2 | 86.0 (12.1) | 88.0 (12.7) |
| - T3 | 86.0 (11.5) | 85.6 (8.3) |
| Median (IQR) WHR at: |  |  |
| - T0 | 0.80 (0.09) | 0.80 (0.13) |
| - T2 | 0.82 (0.08) | 0.81 (0.08) |
| - T3 | 0.84 (0.06) | 0.84 (0.05) |
| Median (IQR) weight (kg) at: |  |  |
| - T0 | 51.5 (14.6) | 51.3 (11.7) |
| - T2 | 55.0 (16.8) | 53.3 (14.7) |
| - T3 | 54.7 (14.4) | 52.9 (11.8) |
| Median (IQR) height (m) at: |  |  |
| - T0 | 1.58 (0.1) | 1.58 (0.1) |
| - T2 | 1.60 (0.1) | 1.60 (0.1) |
| - T3 | 1.61 (0.1) | 1.59 (0.1) |

BMI Body mass index, IQR Interquartile range, HC Hip circumference, WC Waist circumference, WHR Waist to hip ratio, T0 Baseline, T1 Mid-intervention, T2 End of intervention, T3 12-month follow-up

| BMI category, N (%):   - Underweight - Normal - Overweight - Obese | 32 (71%)  258 (80%)  96 (73%)  68 (79%) | 13 (29%)  63 (20%)  35 (27%)  18 (21%) |
| --- | --- | --- |
| BMI z-score, mean (SD) | 0.6 (1.2) | 0.7 (1.2) |
| Baseline physical activity (cpm), mean (SD) | 706 (173) | 716 (234) |
| Group, N (%):   - Intervention - Control | 194 (68%)  263 (87%) | 92 (32%)  40 (13%) |
| School, N (%):   - 1 - 2 - 3 - 4 - 5 - 6 - 7 - 8 - 9 - 10 - 11 - 12 - 13 - 15 - 16 - 17 - 18 - 19 | 17 (65%)  28 (100%)  13 (52%)  30 (83%)  27 (87%)  25 (78%)  24 (62%)  23 (72%)  29 (94%)  20 (67%)  32 (76%)  51 (100%)  33 (94%)  18 (72%)  22 (81%)  21 (72%)  26 (81%)  18 (47%) | 9 (35%)  0 (0%)  12 (48%)  6 (17%)  4 (13%)  7 (22%)  15 (39%)  9 (28%)  2 (7%)  10 (33%)  10 (24%)  0 (0%)  2 (6%)  7 (28%)  5 (19%)  8 (28%)  6 (19%)  20 (53%) |

**Supplementary Table 3:** Baseline (T0) characteristics of pupils who were missing physical activity data at baseline or end of intervention.

BMI Body Mass Index, SD Standard Deviation, cpm Counts Per Minute

**Supplementary Table 4a:** Linear mixed model for the effect of intervention (group) on post-intervention (T2) physical activity (counts per minute, cpm). Adjusted for baseline age, BMI z-scores, and baseline total physical activity.

|  | **Total physical activity (cpm) at end of intervention (T2)** | | |
| --- | --- | --- | --- |
| **Predictors** | **Estimates** | **CI** | ***P*** |
| (Intercept) | 207.40 | -524.14 – 109.35 | 0.199 |
| Group [1] | 33.45 | -21.21 – 88.12 | 0.213 |
| T0 Total physical activity cpm | 0.75 | 0.66 – 0.83 | <0.001 |
| T0 Age | -1.83 | -22.01- 25.68 | 0.880 |
| T0 BMI z score | -0.47 | -11.09 – 12.03 | 0.937 |
| Random Effects  σ^2^  ^Ʈ^00 School ID  ICC  ^N^ School ID | 20208.76  2039.27  0.09  18 |  |  |
| Observations | 435 |  |  |
| Marginal R^2^ / Conditional R^2^ | 0.422 / 0.475 |  |  |

CI Confidence Interval, cpm Counts Per Minute, BMI Body Mass Index

**Supplementary Table 4b:** Adjusted (left; adjusting for baseline age, BMI z-scores, and baseline total physical activity) and unadjusted (right; adjusted only for baseline total physical activity) linear mixed model for the effect of intervention (group) on post-intervention (T2) physical activity (counts per minute, cpm).

|  | **Total physical activity (cpm) at end of intervention (T2)** | | | **Total physical activity (cpm) at end of intervention (T2)** | | |
| --- | --- | --- | --- | --- | --- | --- |
| **Predictors** | **Estimates** | **CI** | ***P*** | **Estimates** | **CI** | ***P*** |
| (Intercept) | 207.40 | -524.14 – 109.35 | 0.199 | 183.25 | 116.88 – 249.61 | <0.001 |
| Group [1] | 33.45 | -21,21 – 88.12 | 0.213 | 34.37 | -19.95 – 88.69 | 0.199 |
| T0 Total physical activity cpm | 0.75 | 0.66 – 0.83 | <0.001 | 0.75 | 0.67 – 0.83 | <0.001 |
| T0 Age | -1.83 | -22.01 – 25.68 | 0.880 |  |  |  |
| T0 BMI z score | -0.47 | -11.09 – 12.03 | 0.937 |  |  |  |
| Random Effects  σ^2^  ^Ʈ^00 School ID  ICC  ^N^ School ID | 20208.76  2039.27  0.09  18 |  |  | 20046.78  2025.58  0.09  18 |  |  |
| Observations | 435 |  |  | 435 |  |  |
| Marginal R^2^ / Conditional R^2^ | 0.422 / 0.475 |  |  | 0.423 / 0.476 |  |  |

CI Confidence Interval, cpm Counts Per Minute, BMI Body Mass Index

**Supplementary Table 5:** The number (%) of pupils meeting recommended physical activity guidelines across groups and timepoints

|  | Overall | | Intervention | | Control | |
| --- | --- | --- | --- | --- | --- | --- |
|  | N | % | n | % | n | % |
| N (%) meeting physical activity guidelines, at T0 baseline:   - Yes - No   [Missing data] | 66  469  [54] | 12  88 | 37  211  [38] | 15  85 | 29  258  [16] | 10  90 |
| N (%) meeting physical activity guidelines, at T1, mid-intervention:   - Yes - No   [Missing data] | 76  442  [89] | 15  85 | 40  195  [51] | 17  83 | 36  229  [38] | 14  86 |
| N (%) meeting physical activity guidelines, at T2, end of intervention:   - Yes - No   [Missing data] | 66  391  [132] | 14  86 | 34  160  [92] | 18  82 | 32  231  [40] | 12  88 |
| N (%) meeting physical activity guidelines, at T3, 12-month follow-up:   - Yes - No   [Missing data] | 55  400  [134] | 12  88 | 22  179  [85] | 11  89 | 33  221  [49] | 13  87 |
